# Chronic administration of ivabradine improves cardiac Ca handling and function in a rat model of Duchenne muscular dystrophy

**DOI:** 10.1038/s41598-025-92927-4

**Published:** 2025-03-15

**Authors:** Jessica Marksteiner, Christopher Dostal, Janine Ebner, Petra Lujza Szabó, Bruno K. Podesser, Simge Baydar, Ana I. A. Goncalves, Anja Wagner, Klaus Kratochwill, Petra Fichtinger, Dietmar Abraham, Isabella Salzer, Helmut Kubista, Elena Lilliu, Benjamin Hackl, Jakob Sauer, Hannes Todt, Xaver Koenig, Karlheinz Hilber, Attila Kiss

**Affiliations:** 1 Department of Neurophysiology and Neuropharmacology, Center for Physiology and Pharmacology, Medical University of Vienna, 1090 Vienna, Austria; 2 Ludwig Boltzmann Institute for Cardiovascular Research at the Center for Biomedical Research and Translational Surgery, Medical University of Vienna, 1090 Vienna, Austria; 3 Core Facility Proteomics, Medical University of Vienna, 1090 Vienna, Austria; 4 Division of Pediatric Nephrology and Gastroenterology, Department of Pediatrics and Adolescent Medicine, Comprehensive Center for Pediatrics, Medical University Vienna, 1090 Vienna, Austria; 5 Center for Anatomy and Cell Biology, Medical University of Vienna, 1090 Vienna, Austria

**Keywords:** Cardiomyocyte calcium handling, Cardiac function, Duchenne muscular dystrophy, Dystrophic rats, Ivabradine treatment, Cell biology, Drug discovery, Cardiology, Diseases, Medical research, Molecular medicine, Pathogenesis

## Abstract

Duchenne muscular dystrophy (DMD), a severe muscle disease caused by mutations in the gene encoding for the intracellular protein dystrophin, is associated with impaired cardiac function and arrhythmias. A causative factor for complications in the dystrophic heart is abnormal calcium (Ca) handling in ventricular cardiomyocytes, and restoration of normal Ca homeostasis has emerged as therapeutic strategy. Here, we used a rodent model of DMD, the dystrophin-deficient DMD^mdx^ rat, to test the following hypothesis: chronic administration of ivabradine (IVA), a drug clinically approved for the treatment of heart failure, improves Ca handling in dystrophic ventricular cardiomyocytes and thereby enhances contractile performance in the dystrophic heart. Intracellular Ca measurements revealed that 4-months administration of IVA to DMD^mdx^ rats significantly improves Ca handling properties in dystrophic ventricular cardiomyocytes. In particular, IVA treatment increased electrically-evoked Ca transients and speeded their decay. This suggested enhanced sarcoplasmic reticulum Ca release and faster removal of Ca from the cytosol. Chronic IVA administration also enhanced the sarcoplasmic reticulum Ca load. Transthoracic echocardiography revealed a significant improvement of cardiac systolic function in IVA-treated DMD^mdx^ rats. Thus, left ventricular ejection fraction and fractional shortening were enhanced, and end-systolic as well as end-diastolic diameters were diminished by the drug. Finally, chronic IVA administration neither significantly attenuated cardiac fibrosis and apoptosis, nor was vascular function improved by the drug. Collectively our findings suggest that long-term IVA administration enhances contractile function in the dystrophic heart by improvement of Ca handling in ventricular cardiomyocytes. Chronic IVA administration may be beneficial for DMD patients.

## Introduction

The heart rate-lowering drug ivabradine (IVA) is clinically approved for the treatment of heart failure and stable angina pectoris^1–4^. Only few studies have so far considered the potential of IVA in the therapy of cardiomyopathies, which emerge in muscular dystrophy patients. Among those, benefiting effects of the drug were evident in the case of a 22-year-old patient with Becker muscular dystrophy, where IVA administration acutely normalized arrhythmia and resolved heart failure, the latter probably by generating a positive inotropic effect^5^. Beneficial effects of long-lasting therapy with IVA (7-year follow-up) in addition to conventional therapy in a patient with Duchenne muscular dystrophy (DMD) were reported by De Benedittis et al. These authors reasoned that, in DMD patients, IVA improves left ventricular function^6^. According with De Benedittis et al., a clinical trial in which the effects of IVA on long-term outcomes in end-stage DMD cardiomyopathy were investigated, suggested a protective role of the drug. Thus, IVA reduced the incidence of acute adverse events, and enhanced left ventricular function^7^. A very recently published clinical study on DMD patients^8^ yielded significantly increased left ventricular ejection fractions after continuous administration of IVA for 6 months and 1 year. Using a rat model of DMD, Tochinai et al. recently reported that 3-months IVA administration ameliorated cardiomyopathy progression^9^. In particular, ventricular function was significantly improved, and cardiac fibrosis development was diminished by trend in IVA-treated compared to untreated dystrophic animals. Taken together, IVA seems to exert beneficial effects on the dystrophic heart both when acutely administered and in the long term. The mechanisms underlying these effects, however, have remained unknown.

We^10^ recently reported cardiac contractile dysfunction associated with disturbed intracellular Ca handling, classical features of the heart in DMD patients^11–14^, in a rat model of the human disease (DMD^mdx^ rats^15^). In a follow-up study, we showed that acute application of IVA significantly improves cardiac function in DMD^mdx^ rats^16^. IVA also acutely improved Ca handling in isolated dystrophic rat ventricular cardiomyocytes, which provided an explanation for drug-induced enhancement of cardiac functionality in DMD^mdx^ rats. In the present study, we used the DMD^mdx^ rat model of DMD to test the following hypothesis: long-term IVA treatment rescues Ca handling defects in dystrophic ventricular cardiomyocytes and thereby improves cardiac function.

## Results

### Long-term treatment of dystrophic rats with IVA rescues impaired Ca handling in ventricular cardiomyocytes

**Fig. 1:**
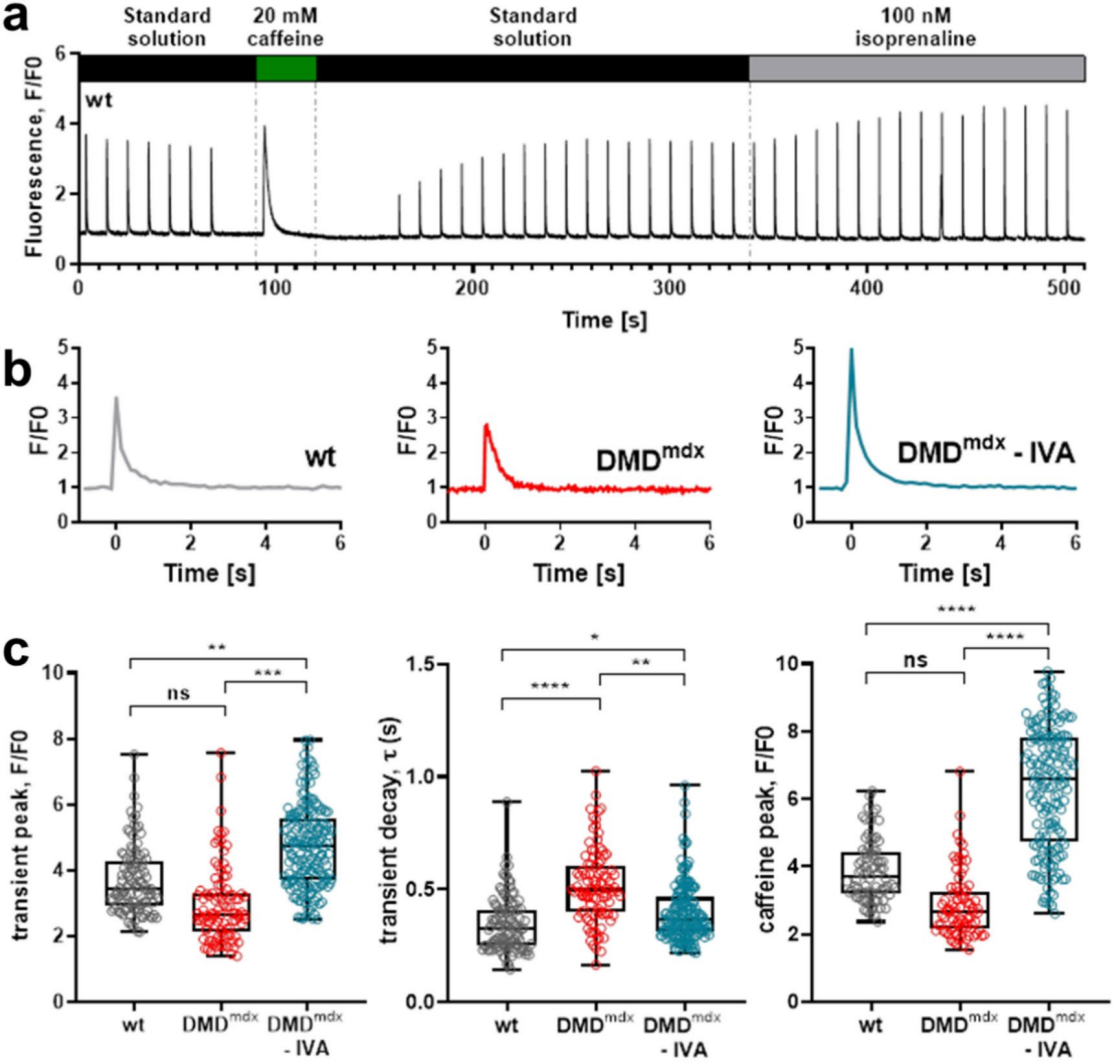
Ca handling properties of ventricular cardiomyocytes derived from from wt, control DMD^mdx^, and IVA-treated DMD^mdx^ rats. (**a**) Original trace example of an intracellular Ca measurement showing the event sequence. Ca transients were first elicited by electrical stimulation at 0.1 Hz frequency. Thereafter, a Ca transient was induced by caffeine application. After caffeine washout, electrical stimulation was started again, and isoprenaline was applied. (**b**) Representative single electrically-evoked Ca transients of a wt, control DMD^mdx^, and IVA-treated DMD^mdx^ myocyte at an enlarged time scale in standard extracellular solution. The decay of the electrically-induced Ca signal following the rapid initial rise was fitted with a single exponential function to derive τ-values. (**c**) *left*: Comparison of mean Ca peak fluorescence relative to baseline (F/F0) between wt, DMD^mdx^ and IVA-treated DMD^mdx^ myocytes. Each data point represents a single cell, and values are expressed as median, interquartile range, and minimum/maximum. [107 cells for wt (5 animals); 97 cells for DMD^mdx^ (6 animals) and 177 cells for IVA-treated DMD^mdx^ (6 animals) myocytes]. (**c**) *middle*: Comparison of the Ca transient decay kinetics in wt, DMD^mdx^ and IVA-treated DMD^mdx^ myocytes. (**c**) *right*: Comparison of mean Ca peak fluorescence, elicited by caffeine application, relative to baseline (F/F0) between wt, DMD^mdx^ and IVA-treated DMD^mdx^ myocytes [82 cells for wt (5 animals); 81 cells for DMD^mdx^ (5 animals) and 167 cells for IVA-treated DMD^mdx^ (6 animals) myocytes]. *p < 0.05, **p < 0.01, ***p < 0,001, ****p < 0,0001; ns, not significant.

We recently demonstrated that acute application of IVA significantly increases the amplitude of electrically-evoked Ca transients in dystrophic ventricular cardiomyocytes derived from DMD^mdx^ rats^16^. This suggested enhancement of Ca release from the sarcoplasmic reticulum (SR) triggered by acute drug application. To study the long-term effects of IVA on Ca handling in the dystrophic heart, we performed intracellular Ca measurements on ventricular cardiomyocytes derived from wild-type (wt), control DMD^mdx^, and 4-months IVA-treated DMD^mdx^ rats (Fig. 1). Figure 1b,c (left) shows that the amplitude of electrically-evoked Ca transients was moderately decreased in DMD^mdx^ compared to wt myocytes. This difference, however, was not statistically significant (p = 0.17). The Ca transient amplitude of cardiomyocytes derived from long-term IVA-treated DMD^mdx^ rats was significantly enhanced when compared to that of myocytes from untreated control DMD^mdx^ and wt rats (Fig. 1b,c, left). This suggested drug-induced enhancement of Ca release from the SR in dystrophic ventricular cardiomyocytes.

The speed of Ca removal from the cytosol after SR Ca release is represented by the decay phase of electrically-evoked Ca transients. Consistent with our earlier work^10^, Fig. 1c (middle) shows that the decay phase of the Ca transient was significantly slowed in DMD^mdx^ compared to wt ventricular cardiomyocytes. Ca transient decay of cardiomyocytes derived from long-term IVA-treated DMD^mdx^ rats was significantly speeded when compared to that of myocytes from untreated control DMD^mdx^ rats, and almost rescued to the wt level (Fig. 1c, middle). This implied drug-induced acceleration of Ca removal from the cytosol after SR Ca release in dystrophic ventricular cardiomyocytes.

The Ca load of the SR can be estimated from the caffeine-induced Ca transient amplitude^17^. Consistent with our earlier work^10^, Fig. 1c (right) shows that the amplitude of caffeine-evoked Ca transients was diminished in DMD^mdx^ compared to wt myocytes. In the present study, however, this difference was not statistically significant (p = 0.21). The amplitude of caffeine-evoked Ca transients of cardiomyocytes derived from long-term IVA-treated DMD^mdx^ rats was significantly enhanced when compared to that of myocytes from untreated control DMD^mdx^ and wt rats (Fig. 1c, right). This suggested drug-induced enhancement of SR Ca load in dystrophic ventricular cardiomyocytes. Here, it was striking that SR Ca load was even significantly higher in IVA-treated DMD^mdx^ compared to wt. This may suggest that IVA treatment does not only prevent pathological “signalling”, but changes the underlying normal physiology.

In our earlier work^10^, we reported normal beta-adrenergic responsiveness of Ca handling in DMD^mdx^ ventricular cardiomyocytes. This was concluded from experiments comparing the acute effects of the beta receptor agonist isoprenaline (100 nM) on electrically-evoked Ca transients in wt and DMD^mdx^ myocytes, which yielded similar Ca transient peak amplitudes in the presence of the drug. Here, consistent with our earlier work, we found that, in the presence of 100 nM isoprenaline, Ca transient amplitude was similar in wt (F/F0: 4.8 ± 0.2, n = 91 cells, 5 animals) and DMD^mdx^ (F/F0: 6.6 ± 0.3, n = 86 cells, 6 animals) myocytes (p = 0.1). Further, Ca transient amplitude in the presence of isoprenaline of cardiomyocytes derived from long-term IVA-treated DMD^mdx^ rats (F/F0: 7.0 ± 0.2, n = 158 cells, 6 animals) was comparable to Ca transient amplitude of untreated control DMD^mdx^ myocytes (p = 0.07). These experiments implied that IVA does not substantially affect beta-adrenergic responsiveness of Ca handling in dystrophic ventricular cardiomyocytes.

### Effects of long-term IVA treatment on the expression of Ca handling proteins in the dystrophic heart

**Fig. 2:**
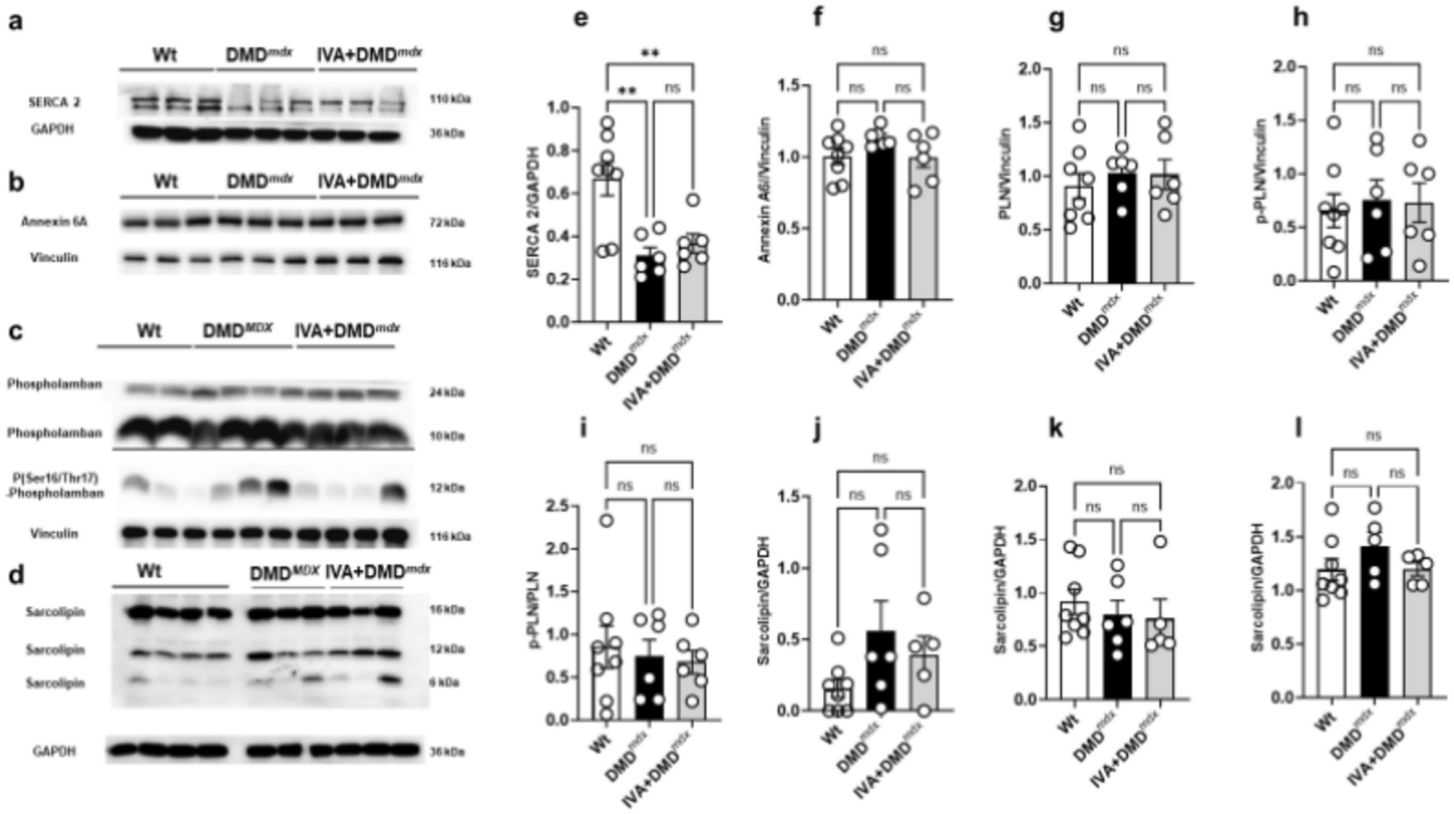
The expression of Ca handling proteins in left ventricular tissue samples. Representative western blots showing SERCA2 (**a**), annexin 6A (**b**) phospholamban (**c**, top), phospho (Ser16/Thr17)-phospholamban (**c**, bottom) and sarcolipin (**d**, monomer and multimer formation) protein levels in left ventricular tissue, which was obtained from wt, DMD^mdx^ and IVA-treated DMD^mdx^ rats. This figure also shows the same lanes after membranes were re-probed for GAPDH or vinculin to demonstrate equal protein loading in each lane. Semi-quantitative analysis of SERCA2 (**e**), annexin 6A (**f**) phospholamban (**g**) and phospho (Ser16/Thr17)- phospholamban (**h**), phospho (Ser16/Thr17)- phospholamban/phospholamban (**i**) and sarcolipin (**j–l**) in left ventricular tissue. Data represent means ± SEM; n = 8 for wt, n = 6 for DMD^mdx^, and n = 6 for IVA-treated DMD^mdx^. Statistical comparisons were carried out using a one-way ANOVA and post-hoc Tukey’s multiple comparison test, **p < 0.01. *DMD* Duchenne Muscular Dystrophy, *GAPDH* glyceraldehyde 3-phosphate dehydrogenase, *IVA* ivabradine, *PLN* phospholamban, *p-PLN* phospho (Ser16/Thr17)-phospholamban, *SERCA2* sarcoplasmic/endoplasmic reticulum Ca ATPase 2, *ns* not significant, *wt* wild-type.

IVA-induced enhancement of Ca release from the SR, speeding up of Ca removal from the cytosol, and enhancement of SR Ca load in dystrophic cardiomyocytes (see above) may be explained by enhanced activity of the SR Ca ATPase (SERCA). SERCA activity enhancement by IVA could be due to drug-induced upregulation of SERCA expression. To test this hypothesis, we performed western blot experiments using left ventricular tissue derived from wt, untreated control DMD^mdx^, and long-term IVA-treated DMD^mdx^ rats. Figure 2a,e shows that SERCA2 protein levels were significantly reduced in DMD^mdx^ compared to wt ventricular tissue. SERCA2 expression in DMD^mdx^ ventricles was not affected by IVA treatment, suggesting that SERCA activity enhancement by IVA cannot simply be explained by increased SERCA protein levels. Enhancement of Ca handling in dystrophic cardiomyocytes by IVA could alternatively be explained by drug-induced suppression of annexin A6 expression. Overexpression of this protein inhibits SR Ca release^18,19^, and its knockdown speeds Ca removal from the cytosol^20,21^. Figure 2b,f shows that annexin A6 protein levels were similar in wt and DMD^mdx^ ventricles, and also unaffected by IVA treatment, suggesting that annexin A6 expression is not suppressed by the drug. Another potential cause for SERCA activity enhancement by IVA would be diminished inhibition of SERCA by phospholamban in the presence of the drug. Phospholamban expression was similar in wt and DMD^mdx^ ventricles, and also unaffected by IVA treatment in the latter (Fig. 2c,g). Further, no difference existed between serine 16 and threonine 17 phosphorylation of phospholamban and the ratio phospho (Ser16/17)-phospholamban/phospholamban in ventricular tissue from wt, control DMD^mdx^ and IVA-treated DMD^mdx^ rats (Fig. 2c,h,i). Together, these findings suggested that long-term IVA presence did not affect the expression and phosphorylation status of the SERCA-inhibitory protein phospholamban. Finally, the expression of sarcolipin, another SERCA inhibitor, was similar in wt and DMD^mdx^ ventricles, and also unaffected by IVA treatment in the latter (Fig. 2d,j–l).

With the intention to provide additional information about the expression of Ca regulatory proteins in wt, DMD^mdx^, and IVA-treated DMD^mdx^ hearts, we performed a proteomics study targeted at “calcium regulation in cardiac cells” and “key SR Ca-buffering proteins”, the results of which are given in Supplemental Figs. 1 and 2, and Supplemental Table 1. In summary, besides a few significant differences between wt and DMD^mdx^, no significant differences in the expression of Ca regulatory proteins could be observed between DMD^mdx^ and IVA-treated DMD^mdx^.

### Effects of long-term IVA treatment on the function of the dystrophic heart

**Fig. 3:**
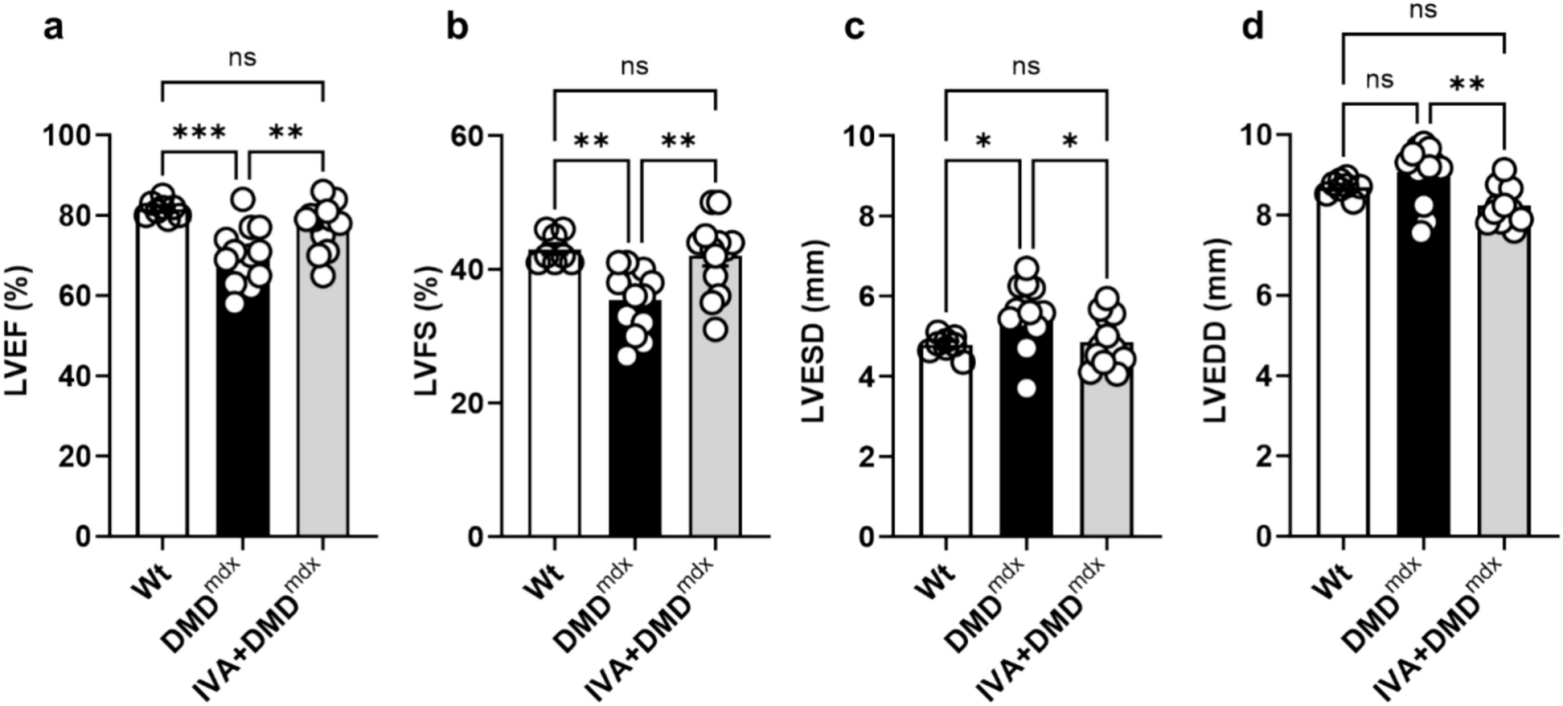
Heart function and dimensions assessed by transthoracic echocardiography. Comparison of echocardiography parameters between wt, DMD^mdx^ and IVA-treated DMD^mdx^ rats. All parameters were measured for at least three successive cardiac cycles. LVEF: left ventricular ejection fraction (**a**); LVFS: left ventricular fractional shortening (**b**); LVESD: Left ventricular end-systolic diameter (**c**); LVEDD: left ventricular end-diastolic diameter (**d**). Data represent means ± SEM; n = 8 wt rats; n = 13 DMD^mdx^ rats, and n = 13 IVA-treated DMD^mdx^ rats. Statistical comparisons were carried out using a one-way ANOVA and post-hoc Tukey’s multiple comparison test, *p < 0.05, **p < 0.01 and ***p < 0.001. DMD: Duchenne Muscular Dystrophy, IVA: ivabradine; ns: not significant, and wt: wild-type.

In order to test whether the IVA-induced enhancement of Ca handling properties in dystrophic ventricular cardiomyocytes resulted in an improvement of cardiac functionality, heart functional parameters and dimensions were assessed by transthoracic echocardiography. According to our previous study^10^, we found that left ventricular (LV) ejection fraction (LVEF) and fractional shortening (LVFS) were significantly reduced in DMD^mdx^ compared to wt rats (Fig. 3a,b). In addition, LV end-systolic diameter (LVESD) was enlarged in dystrophic animals (Fig. 3c). In line with Tochinai et al.^9^, chronic IVA administration to DMD^mdx^ rats rescued the named parameters to wt levels (Fig. 3a–c). IVA also significantly decreased LV end-diastolic diameter (LVEDD) in DMD^mdx^ rats (Fig. 3d). In contrast to the present study, this parameter (similar in wt and DMD^mdx^ rats both in the present study and in Tochinai et al.) was unaffected by IVA treatment in Tochinai et al. Together our data suggested that chronic IVA administration effectively counteracts impaired LV function and LV dilation in the heart of dystrophic DMD^mdx^ rats.

### Effects of long-term IVA treatment on cardiac histopathology

**Fig. 4:**
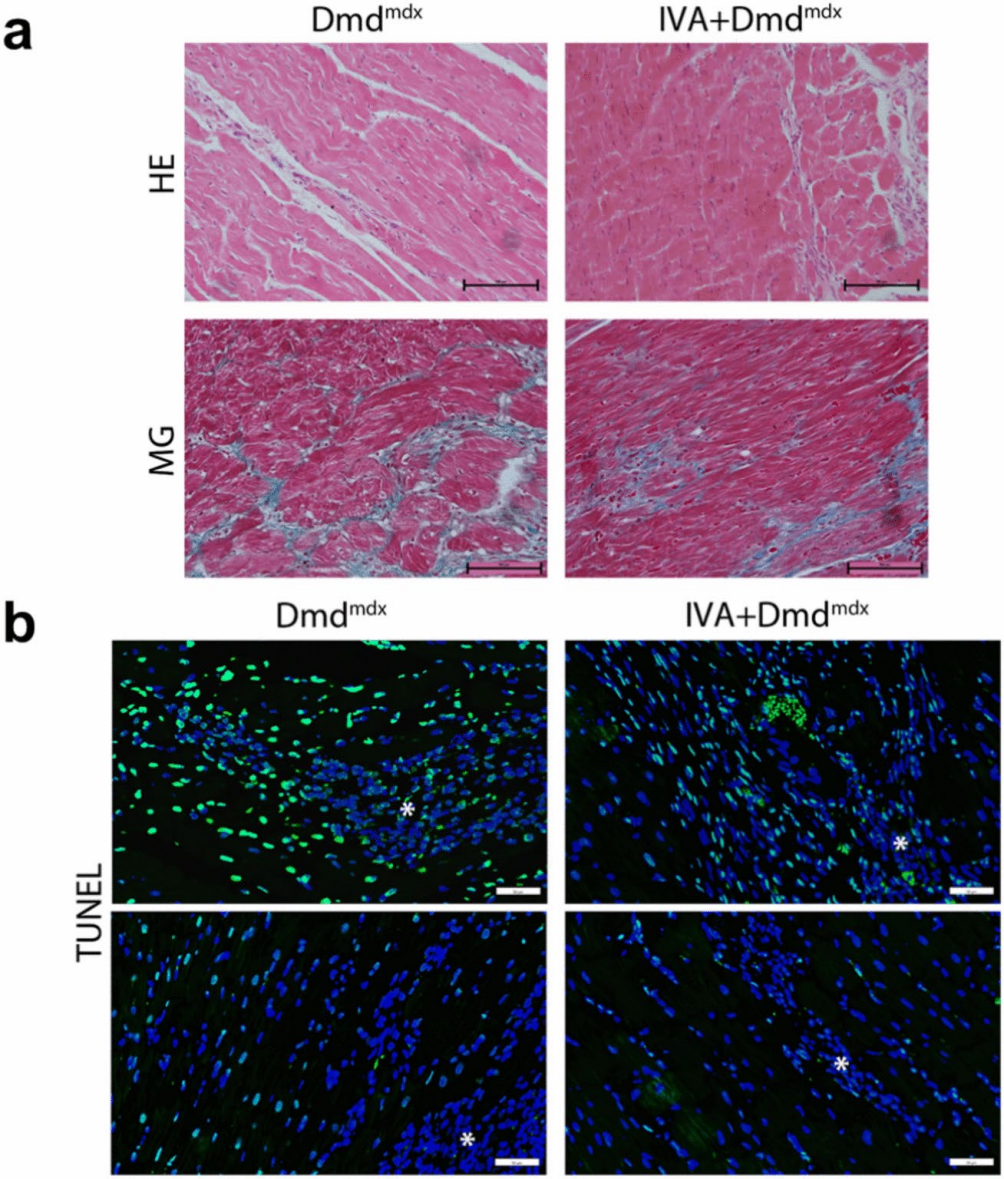
The impact of chronic ivabradine administration on cardiac fibrosis and apoptosis. (**a**) Representative pictures of haematoxylin & eosin (HE)-stained cardiac sections, and representative images of myocardial fibrosis stained with the Masson trichrome method (MG). Cardiac sections derived from untreated control DMD^mdx^ rats (left) and IVA-treated DMD^mdx^ rats (right) are compared. (**b**) Representative photographs of TUNEL staining in cardiac sections of untreated DMD^mdx^ (left) and IVA-treated DMD^mdx^ (right) rats. This assay was performed to assess the degree and distribution of apoptotic cells in normal and fibrotic/remodelled heart tissue. Green dots represent apoptotic cells, resulting from the overlay of TUNEL-stained and DAPI-counterstained nuclei. *Areas with inflammatory cells which are TUNEL-negative. Blue dots represent non-apoptotic cells stained with DAPI only. Scale bar = 50 µm (200 × magnification). *DAPI* 4′,6-diamidino-2-phenylindole, *DMD* Duchenne Muscular Dystrophy, *IVA* ivabradine.

Cardiac fibrosis is a major contributor to cardiac dysfunction in DMD^22,23^. In line with that, we have previously demonstrated a significant amount of cardiac fibrosis in association with a decline in cardiac function in DMD^mdx^ rats^10^. In the present study, we evaluated the histopathology in left ventricular tissue specimens using haematoxylin and eosin (HE) staining and Masson Goldner (MG) staining. In untreated control DMD^mdx^ rats, the myocardium exhibited significant inflammatory cell infiltration and collagen deposition, leading to severe myocardial structural changes (Fig. 4a). A similar picture was observed in the IVA-treated DMD^mdx^ rat group. A hallmark of dystrophin-deficient muscle is the dysregulation of Ca homeostasis (e.g. increased cytosolic Ca levels) leading to the activation of proteases and DNA fragmentation. In line with that, positivity to TUNEL staining is often spotted in DMD as a maker for the loss of skeletal and cardiac myocytes^24,25^. Therefore, we assessed TUNEL-staining in left ventricular specimens, and selected two distinguished areas within the cardiac tissue: normal tissue and fibrotic/remodelled areas; TUNEL staining (Fig. 4b) showed few TUNEL-positive cells in normal tissue areas, and the inflammatory cells were TUNEL-negative in both groups. In fibrotic/remodelled areas, inflammatory cells were also TUNEL-negative, while numerous TUNEL-positive cells were present in the surrounding fibrotic tissue (Fig. 4b). A similar pattern was observed in IVA-treated animals showing a tendency for reduced fibrotic areas and fewer TUNEL-positive cells. These findings accord with Tochinai et al., who reported a trend toward ameliorated myocardial fibrosis after 3 months of IVA administration to dystrophic rats^9^.

### Effects of long-term IVA treatment on vascular function in dystrophic rats

**Fig. 5:**
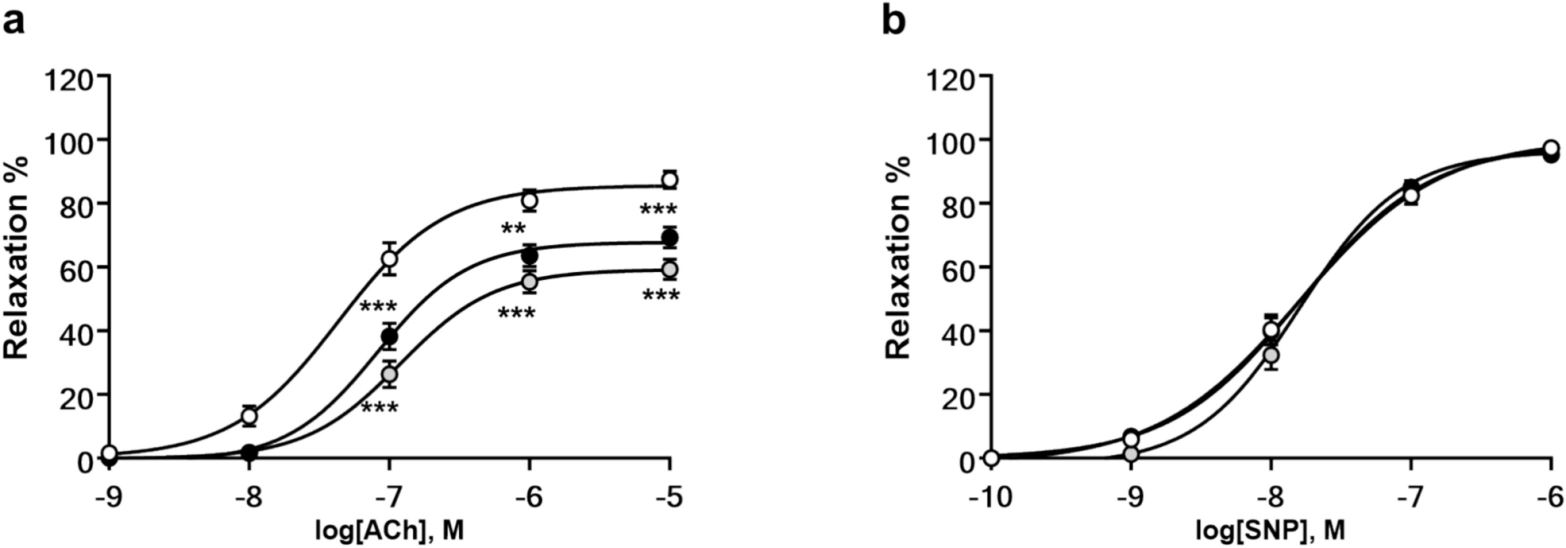
Vascular function analysis. Endothelium-dependent (**a**) and endothelium-independent (**b**) relaxation curves from isolated rings of abdominal aortas from wt (empty circles), DMD^mdx^ (black circles), and IVA-treated DMD^mdx^ (grey circles) rats in response to increasing concentrations of acetylcholine (ACh) and sodium nitroprusside (SNP), respectively. Data represent means ± SEM; n = 17 for wt, n = 15 for DMD^mdx^, and n = 15 for IVA + DMD^mdx^ aorta segments. Statistical comparisons were carried out using a repeated measure-ANOVA and post-hoc Tukey’s multiple comparison test, **p < 0.01 and ***p < 0.001 vs wt. *DMD* Duchenne Muscular Dystrophy, *IVA* ivabradine, *wt* wild-type.

Chronic IVA administration is associated with a reduction in aortic stiffness and endothelial dysfunction^26,27^. Consequently, IVA may improve vascular dysfunction in DMD. In our previous study^10^, we demonstrated that vascular endothelial function was impaired in 9-months-old DMD^mdx^ rats when compared to age-matched wt rats. Here, we elucidated whether IVA treatment improves vascular function in isolated aortic segments from dystrophic rats. Relaxation of aortic rings was tested in myography experiments at an animal age of 9 months. Endothelium-dependent vascular response to increasing concentrations of acetylcholine (ACh) was significantly impaired in DMD^mdx^ rats when compared with wt animals (Fig. 5a). IVA treatment of DMD^mdx^ rats did not improve vasorelaxation. Endothelium-independent relaxation induced by sodium nitroprusside (SNP) did not significantly differ between wt, untreated control DMD^mdx^, and IVA-treated DMD^mdx^ rats (Fig. 5b). These data suggested that IVA treatment does not affect vascular endothelial function in dystrophic rats.

## Discussion

Here we show that long-lasting administration of IVA improves cardiac Ca handling in a rat model of DMD. In particular, the drug increased the amplitude and speeded the decay of electrically-evoked Ca transients. This suggested enhanced SR Ca release and speeded removal of Ca from the cytosol in the presence of IVA. IVA also increased caffeine-induced Ca transients suggesting enhancement of SR Ca load. In accordance with Tochinai et al.^9^, we also report significant improvement of cardiac function in dystrophic rats as a consequence of chronic IVA administration.

### IVA-induced improvement of Ca handling in cardiomyocytes may explain enhanced function in the dystrophic heart

To the best of our knowledge, the present study is the first to show that long-term IVA administration improves Ca handling in the dystrophic heart. In accordance, however, several other authors have previously reported beneficial effects of chronic IVA application on cardiomyocyte/cardiac Ca handling in rodent models of heart failure. Among those, Navaratnarajah et al. showed that 4-week IVA treatment increased electrically-evoked Ca transients and enhanced SR Ca load in cardiomyocytes from a rat heart failure model^28^. Moreover, chronic IVA application altered the expression (FKBP12/12.6^29^) and phosphorylation (phospholamban^30^) of major Ca regulatory proteins in failing rodent hearts. These effects were in each case interpreted by the authors as beneficial for cardiac Ca handling and function. Together, the named studies imply that chronic IVA application improves impaired cardiac Ca handling in the failing heart. The present study adds dystrophic cardiomyopathy to the range of cardiac diseases with impaired Ca handling, which could be ameliorated by long-term IVA treatment.

Studies on DMD animal models have revealed cardiac functional impairments and significant Ca handling abnormalities in dystrophic cardiomyocytes^10,17,31–34^. Typically, electrically-evoked intracellular Ca transients of dystrophic myocytes have decreased amplitudes (e.g.^10,32^; observed only in tendency in the present study), and their decaying phase is significantly decelerated (e.g.^10,17,34^; the present study). This implies diminished SR Ca release and slowed removal of Ca from the cytosol, respectively. In the failing mammalian heart, impaired Ca handling in ventricular cardiomyocytes weakens contractility and, consequently, cardiac function^35–38^. Particularly, diminished Ca release from the SR diminishes cardiac systolic function, and slowed Ca removal from the cytosol impairs diastolic function. These well-established facts ascribed to the failing heart, and the findings of the present study, lead us to propose that, in the dystrophic rat heart, chronically administered IVA rescues cardiac dysfunction by improvement of Ca handling. Thus, enhanced SR Ca release and speeded removal of Ca from the cytosol in the presence of IVA may improve systolic and diastolic function, respectively. In the present study, IVA-induced enhancement of systolic function was implied by significantly increased LVEF and LVFS. In addition, decreased LVESD in the presence of IVA was observed. The latter finding suggests a positive inotropic effect of the drug in the dystrophic heart.

Chronic IVA administration to DMD^mdx^ rats had no significant attenuating effect on cardiac fibrosis and apoptosis. Therefore, drug-induced mitigation of histopathology in the dystrophic heart may not have considerably contributed to the observed enhancement of function in the IVA-treated dystrophic heart. In accordance with Tochinai et al.^9^, however, the present study revealed at least a trend towards diminished fibrosis and apoptosis as consequence of chronic IVA administration to DMD^mdx^ rats. Here, it is currently unclear if IVA directly affects adverse myocardial remodeling, or if IVA-induced hemodynamical improvements lead to secondary attenuation of remodeling.

Finally, an additional mechanism by which IVA may enhance function in the dystrophic heart is improvement of cardiac energetics. In preclinical models of acute coronary syndromes, improved cardiac energetics (e. g. increased mitochondrial ATP production and Ca retention capacity) contributed to the cardioprotective effects of IVA^39,40^.

### Potential mechanism(s) behind Ca handling enhancement by long-term IVA treatment

Bigger electrically-evoked Ca transients in dystrophic ventricular cardiomyocytes in the presence of IVA suggest enhanced Ca release from the SR via ryanodine receptors. This may originate from increased ryanodine receptor activity and/or from increased SR Ca load. In the present study, we report strongly enhanced SR Ca load in cardiomyocytes from IVA-treated DMD^mdx^ rats. This may at least partly explain bigger Ca transient amplitudes in the presence of the drug. Because IVA also significantly speeded the decay phase of the electrically-evoked Ca transient, we propose that the activity of SERCA was increased by the drug, which may be causal for enhancement of SR Ca load. We recently showed that, similar as chronic application, acute application of IVA to dystrophic rat ventricular cardiomyocytes increased the Ca transient amplitude. In contrast, however, acute application failed to speed Ca transient decay^16^. Together with the present study this suggests that chronic, but not acute IVA administration, enhances SERCA activity. In order to shed light on the mechanism(s) underlying the modulation of Ca handling properties by IVA, we measured protein levels of two major Ca handling proteins, SERCA2 and its regulatory protein phospholamban, in ventricular tissue by western blotting. These experiments suggested that SERCA2 and phospholamban expression were not affected by long-term IVA presence. Expression changes of these two proteins, thus, cannot explain IVA-induced enhancement of SERCA activity in the dystrophic heart. In accordance with the present study, using a rabbit model of heart failure, Couvreur et al. showed that chronic IVA administration had no effect on SERCA2 and phospholamban expression in myocardial samples^29^. Reil et al., using a mouse model of heart failure, reported that 4-week IVA treatment of mice resulted in significant threonine 17 phosphorylation of phospholamban in ventricular tissue, potentially enhancing SERCA activity and accelerating myocardial relaxation^30^. A similar effect of IVA in the dystrophic heart would have provided a potential explanation for the enhancement of SERCA activity by the drug. However, we found serine 16 and threonine 17 phosphorylation of phospholamban to be similar in ventricular tissue from control DMD^mdx^ and IVA-treated DMD^mdx^ rats (Fig. 2h), thereby ruling out this anticipated mechanism. Finally, our western blot experiments have also excluded regulation of annexin A6 expression as potential mechanism for Ca handling enhancement by long-term IVA treatment. The same was true for the expression of the SERCA2 inhibitor sarcolipin, which was unaffected by IVA.

Our proteomics study basically confirmed the results of the above-described western blot investigations in that the expression of SERCA2 (Atp2a2) and annexin A6 (Anxa6) was not significantly modulated by IVA-treatment. Both phospholamban and sarcolipin could not be detected with the applied proteomics approach. In contrast to the western blot data, the proteomics data did not reveal a significant down-regulation of SERCA2 in DMD^mdx^ versus wt ventricular samples (Atp2a2 in Supplemental Table 1). Moreover, annexin A6 expression was similar in wt and DMD^mdx^ relating to the western blot data, but significantly increased in the latter based on the proteomics data. These discrepancies represent a limitation of the present study. Finally, chronic IVA application significantly enhanced the expression of the ryanodine receptor stabilizing protein FKBP12/12.6 in a rodent model of heart failure^29^. This was interpreted by the authors as beneficial for cardiac Ca handling and function. Our proteomics data, however, suggested that FKBP12/12.6 (Fkbp1a) expression in DMD^mdx^ ventricles was unaffected by IVA treatment and can therefore not account for IVA-induced enhancement of Ca handling in dystrophic cardiomyocytes.

Taken together, from our extensive protein expression studies we reason that chronic administration of IVA may improve cardiac Ca handling in the dystrophic heart via another mechanism (or other mechanisms) than simply by significant modulation of the expression levels of Ca regulatory proteins. Here, IVA-induced changes in the functional regulation of Ca regulatory proteins are conceivable. Further studies are needed to clarify the actual as yet unknown mechanism(s).

### Expectable effects of chronic IVA application on the dystrophic human heart

DMD^mdx^ rats show reduced cardiac functionality consistent with dilated cardiomyopathy development^10,15^. Moreover, intracellular Ca handling in DMD^mdx^ ventricular cardiomyocytes is significantly impaired^10^. Cardiac malfunction, development of a dilated cardiomyopathy^12,13^, and impaired Ca handling^11,14,41^ are also features of the dystrophic human heart, whereby striking similarities to the DMD^mdx^ rat heart are obvious. In particular, electrically-induced Ca transient amplitudes were diminished, and Ca transient decay kinetics was decelerated both in DMD^mdx^ rat cardiomyocytes^10^ and in induced pluripotent stem cell-derived cardiomyocytes from DMD patients^11,14^. Taken together, these studies suggest that the Ca handling abnormalities in the heart of DMD^mdx^ rats closely mimic those present in the heart of DMD patients. We therefore speculate that the IVA-induced rescue of impaired Ca handling we report herein for the dystrophic rat heart will also work in the human DMD heart. Thus, IVA-induced enhancement of SERCA activity in cardiomyocytes may improve cardiac function and thereby be beneficial for DMD patients. This accords with clinical trials on DMD patients reporting enhanced left ventricular function as result of long-term administration of IVA^7,8^.

Rescue of abnormal Ca handling in the dystrophic heart by chronic IVA administration, thus, emerges as a promising strategy for DMD pharmacotherapy.

## Methods

### Ethics statement

The study is in accordance with the rules of the Animal Welfare Committee of the Medical University of Vienna. The experimental protocols and procedures were approved by the Austrian Science Ministry (ethics vote number: BMWFW-66.009/0175-WF/V/3b/2015).

### Animals and IVA treatment procedure

Male wild-type (wt) and dystrophin-deficient DMD^mdx^ Sprague Dawley rats^15^ originated from INSERM-CRTI UMR 1064 (Nantes). Genotyping of the rats was performed using standard PCR assays as previously described^15^. IVA (Ivabradine-HCl, I0847, TCI Deutschland GmbH) administration to DMD^mdx^ rats was performed via the drinking water in a dose of 10 mg·kg^−1^·day^−1^ as in other rodent studies, which have previously tested long-term effects of IVA on the heart^28,29,42,43^. The drug treatment started at an animal age of 5 months. At this age, DMD^mdx^ rats show a cardiomyopathy with significantly impaired heart function^10^. IVA treatment lasted for 4 months and resulted in a significant heart rate reduction (measured before animal scarification: 249 ± 18 bpm in untreated control DMD^mdx^ rats versus 216 ± 17 bpm in IVA-treated DMD^mdx^ rats; p < 0.01; n = 6 and 7, respectively). The control DMD^mdx^ rat group was given the same amount of drinking water. Age-matched (9-months-old) wt Sprague Dawley rats were used as healthy controls.

### Isolation of ventricular cardiomyocytes

Male wt and DMD^mdx^ rats (n = 5 wt and n = 12 DMD^mdx^ animals) at the age of 9 months were anesthetized using isoflurane (2%, inhalation) and killed by cervical dislocation. Cardiomyocytes were isolated from the ventricles of their hearts using a Langendorff setup. The details of the applied myocyte isolation procedure from rodent hearts can be found in our previously published studies^10,44^. Briefly, rat hearts were rapidly excised, and a cannula was inserted into the aorta for retrograde perfusion with Ca-free solution containing 0.17 mg/mL Liberase TH (Roche) at 37 °C for 18 min. Thereafter, the ventricles were cut into pieces and incubated on a shaker at 37 °C, and Ca concentration was increased to 150 μM over 30 min in four steps. Pieces of digested ventricular tissue were then triturated to release cardiomyocytes. After a centrifugation step, the myocytes were resuspended in minimum essential medium (MEM)-alpha (Sigma), containing ITS media supplement (Sigma) diluted (1:100), 2 mM l-glutamine, 100 U/mL penicillin, 0.1 mg/mL streptomycin, and 17 µM blebbistatin (Sigma). Myocytes were finally plated on Matrigel (Becton Dickinson)-coated culture dishes.

### Intracellular Ca measurements

Ca transients were recorded from isolated rat ventricular wt and DMD^mdx^ cardiomyocytes at room temperature by using a similar protocol as described in our previous study^34^. In brief, myocytes preloaded with the cell membrane-permeable Ca-sensitive fluorescent dye Fluo-4 AM (Thermo Fisher Scientific) were bathed in standard extracellular solution containing (in mM) 140 NaCl, 4 KCl, 2 CaCl_2_, 2 MgCl_2_, 5 HEPES, and 5 glucose (pH adjusted to 7.4 with NaOH). In order to elicit Ca transients, electrical stimulation was performed at 0.1 Hz via platinum electrodes placed in the bath in 3.5 cm glass bottom dishes. To elicit caffeine-induced sarcoplasmic reticulum (SR) Ca release, bath solution containing 10 mM caffeine was applied via an OctaFlow II perfusion system (ALA Scientific Instruments). To assess the effect of beta-adrenergic stimulation on electrically-evoked Ca transients, at the end of each experiment, the cells were superfused with bath solution containing 100 nM isoprenaline. The precise event sequence of an intracellular Ca measurement is shown in Fig. 1a. Dye fluorescence signals were acquired using a confocal microscope system (Nikon A1R +). Fluorescence peaks upon electrical stimulation (single pulses) or with caffeine were evaluated relative to baseline fluorescence prior to stimulation (F0). To evaluate the duration of electrically-evoked Ca transients, a single exponential function was fitted to the decaying fluorescence to obtain the respective time constants (τ-values). The use of a single exponential function for fitting revealed mean r^2^ values between 0.97 and 0.98. Always the average amplitude and duration of the last five Ca transients prior to solution change (in steady-state) yielded a single data point.

### Western blotting

Total protein was extracted from rat left ventricular tissue using a RIPA buffer (Sigma, 0278) as described previously^23^. A quantity of 30 µg of total protein was loaded into 10% or 15% SDS-PAGE gels, according to the size of the protein of interest. The proteins were transferred to polyvinylidene difluoride (PVDF) membranes, and the membrane was blocked using EveryBlot Blocking Buffer (12010020, BioRad) for 15 min. Then, the membranes were incubated overnight at 4 °C with the following primary antibodies: mouse antibody to SERCA2 ATPase clone 2A7-A1 (ThermoFisher, MA3-919, 1:1000), mouse antibody to vinculin clone hVin-1 (Sigma, V9131, 1:2000), mouse antibody to annexin A6 clone 4C4 (ThermoFisher, MA5-49264, 1:1000), rabbit antibody to phospholamban clone EPR 21897 (abcam, 219626, 1:1000), rabbit antibody to phospho-phospholamban (Ser16/Thr17) (Cell Signalling, 8496, 1:1000), mouse antibody to GAPDH clone ZG003 (Invitrogen, 39-8600, 1:2000), and rabbit antibody to sarcolipin (MERCK, ABT13,1:500). The following day, the membranes were incubated for 1 h at room temperature with the respective secondary antibodies; peroxidase-conjugated AffiniPure Goat Anti-Rabbit IgG (H + L) (111–035-003, Jackson Immuno Research) and Peroxidase-conjugated AffiniPure Goat Anti-Mouse IgG + IgM (H + L) (115-035-044, Jackson Immuno Research). Detection was performed using a ChemiDoc XRS + instrument (BioRad) and a SuperSignal West Femto Maximum Sensitivity Substrate solution (ThermoFisher, 34094) for chemiluminescence detection. The image quantification was conducted using ImageJ.

### Transthoracic echocardiography

Male wt and DMD^mdx^ rats aged 9 months were anesthetized via intraperitoneal injection of a xylazine (4 mg/kg; Bayer) and ketamine (100 mg/kg; Dr. E. Gräub AG) mixture. They were then intubated with a 14-gauge tube and ventilated at a tidal volume of 9 ml/kg and a rate of 75–85 strokes per minute. Rectal temperature was monitored and maintained at 37.5–38.5 °C using a heated operating table. Heart rate (HR) was determined from the electrocardiogram signal. Transthoracic echocardiography was performed to measure end-diastolic (ED) and end-systolic (ES) left ventricular (LV) diameters (D) using M-mode tracing. Left ventricular ejection fraction (LVEF) and fractional shortening (FS) were calculated with the ACUSON SC2000 Ultrasound System (Siemens Healthineers, 4V1c probe) as described previously^45^. LVEDD, LVESD, and LVEF were determined through three independent measurements at the midpapillary short-axis view.

### Cardiac fibrosis assessment

Formalin-fixed paraffin-embedded tissue sections from the anterior wall of left ventricular tissue were haematoxylin and eosin (HE) stained as described previously^10^. The extent of fibrosis in cardiac muscle sections was visualized by Masson Goldner (MG) staining (MG staining kit, Sigma-Aldrich/Merck, Darmstadt, Germany). Images were acquired by microscopy (Olympus VS120 Virtual Slide Microscope System; Olympus, Tokyo, Japan) and captured by digital camera (AVT PIKE F-505C VC 50; Allied Vision Technologies, Stadtroda, Germany).

### TUNEL staining

The Terminal deoxynucleotidyl transferase-mediated dUTP nick end labelling (TUNEL) assay for detection of apoptotic cells on paraffin-embedded (IHC-P) samples was performed using the in situ cell death detection kit, fluorescein (Roche, Mannheim, Germany) according to the manufacturer’s instructions. Briefly, proteinase K was diluted in PBS to a working concentration of 20 µg/ml and applied to the slides, which were then incubated at room temperature. The TUNEL reaction mixture was freshly prepared by combining enzyme solution with label solution, following the kit instructions. Slides were rinsed with PBS, the TUNEL reaction mixture was applied, and the slides were incubated in a humidified atmosphere at 37 °C in the dark. After incubation, the slides were washed with PBS, counterstained with DAPI, rinsed with PBS and ddH_2_O, and mounted with Mowiol (Sigma-Aldrich, Darmstadt, Germany). Fluorescence of heart tissue was detected by fluorescence microscopy (Slidescanner Olympus VS120, Hamburg, Germany) using OLYMPUS-OlyVIA software.

### Vascular function assessment

Vascular function was assessed in isolated aortic segments as described previously^10^. Briefly, segments of the aorta abdominalis of rats were isolated and placed into cold and oxygenated (5% CO_2_ and 95% O_2_) Krebs buffer containing 119 mmol/l NaCl, 4.7 mmol/l KCl, 2.5 mmol/l CaCl_2_, 1.17 mmol/l MgSO_4_, 20 mmol/l NaHCO_3_, 1.18 mmol/l KH_2_PO_4_, 0.027 mmol/l EDTA and 10.5 mmol/l glucose, resulting in pH 7.4. Isolation was performed on ice, and hereby perivascular fat tissue was removed, and the lumen of the vessel was gently perfused using Krebs buffer to remove remaining blood. Afterwards 1.5–2 mm segments were cut and mounted onto a multi chamber isometric myograph system (Model 620 M, Danish Myo Technology, Aarhus, Denmark), which was filled with 6 ml of heated (37 °C) and oxygenated Krebs buffer. Resting tension of the vessels was normalized to 100 mm Hg or 13.3 kPa using DMT normalization settings and La Place’s Law. After 30 min of equilibration time, a reference contraction was elicited by hyperkalemic (124 mM, KCl) solution to visualize maximum contraction of the vessels. After 3 min, the solution was changed again to Krebs buffer. 15 min after baseline was reached, precontraction of the segments was performed using increasing concentrations of phenylephrine (Phe, 1 nM–10 µM; Sigma-Aldrich). Endothelial-dependent relaxation was tested using acetylcholine (ACh, 1 nM–10 µM; a nitric oxide-dependent vasodilator; Sigma-Aldrich). The chambers were washed again with Krebs buffer and were allowed to equilibrate at baseline for 15 min. Afterwards data on endothelial-independent relaxation was acquired using constriction with 1 nM Phe and increasing doses of sodium nitroprusside (SNP, 0.1 nM–1 µM; a nitric oxide-independent vasodilator; Merck), respectively. The data was continuously recorded using the software program LabChart Pro (ADInstruments).

### Proteomic analysis

Details of the applied quantitative proteomics approach based on tandem mass tag (TMT) labelling and two-dimensional high pH offline fractionation LC–MS are provided in supplemental methods and supplemental figure legends.

### Statistical analyses

For the results of the intracellular Ca measurements, statistical comparisons were carried out using a nested analysis respecting the hierarchical data structure (measurements of n cells from m animals) detailed in Sikkel et al.^46^. For testing the results of protein expression in cardiac tissue and cardiac function one-way ANOVA was used, followed by Tukey’s multiple comparison test. Vascular relaxation in response to ACh or SNP was expressed as percentage of contraction induced by Phe. The statistical comparison between the relaxation and contraction responses was assessed using two-way analysis of variance for repeated measures. A p value < 0.05 was considered significantly different. Data were expressed as means ± SEM.

## Supplementary Information

Supplementary Information 1.

Supplementary Information 2.

Supplementary Table 1.

Supplementary Figure 1.

Supplementary Figure 2.

## Data Availability

The data sets used and/or analysed during the current study will be made available upon reasonable request directed to the corresponding author.
