# Supplementary figures and images for: Chronic administration of ivabradine improves cardiac Ca handling and function in a rat model of Duchenne muscular dystrophy
Source: Sci Rep. 2025 Mar 15;15:8991. doi: 10.1038/s41598-025-92927-4 (PMC11910634; doi:10.1038/s41598-025-92927-4)

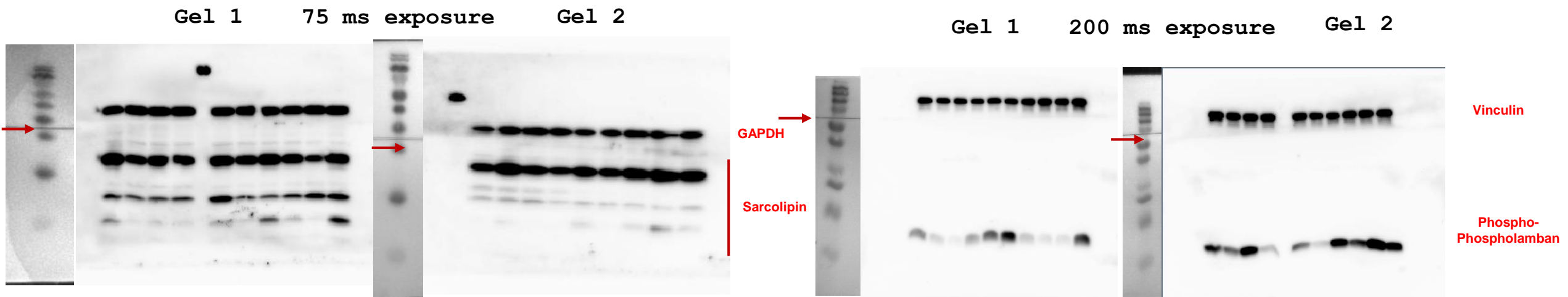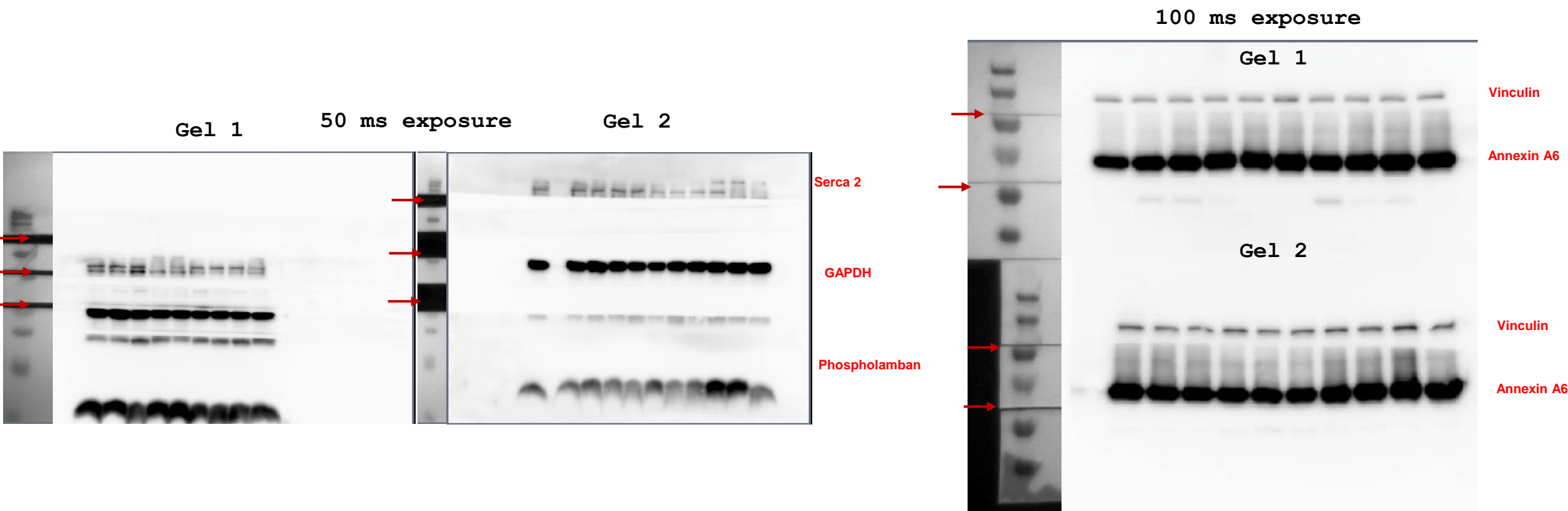

Supplement: Supplementary file 1 — Supplementary Information 1. [file 41598_2025_92927_MOESM1_ESM.pdf]

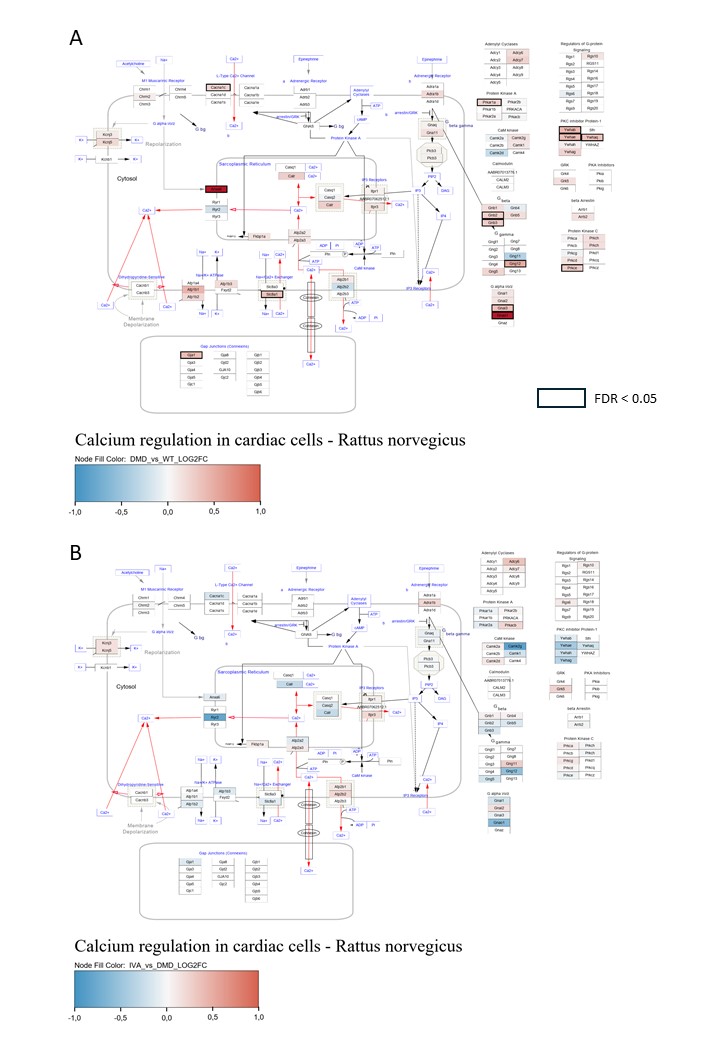

Supplement: Supplementary file 4 — Supplementary Figure 1. [file 41598_2025_92927_MOESM4_ESM.jpg]

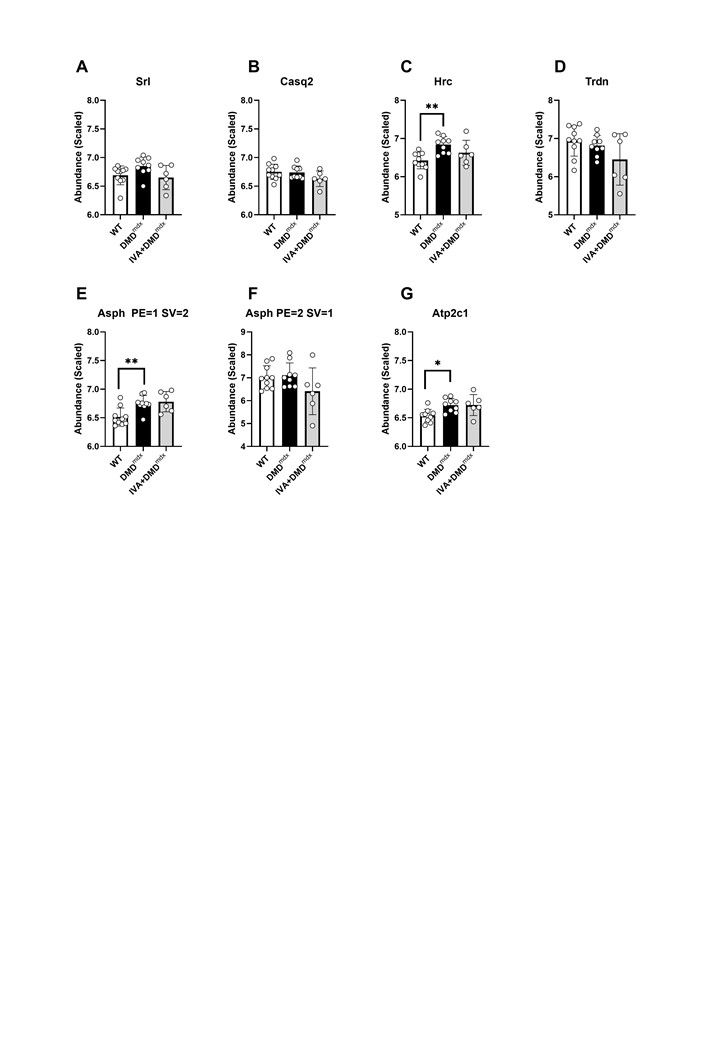

Supplement: Supplementary file 5 — Supplementary Figure 2. [file 41598_2025_92927_MOESM5_ESM.jpg]
