# Supplementary material for: Chronic administration of ivabradine improves cardiac Ca handling and function in a rat model of Duchenne muscular dystrophy
Source: Sci Rep. 2025 Mar 15;15:8991. doi: 10.1038/s41598-025-92927-4 (PMC11910634; doi:10.1038/s41598-025-92927-4)
Supplement: Supplementary file 2 — Supplementary Information 2. [file 41598_2025_92927_MOESM2_ESM.docx]

**Supplemental methods**

*Sample preparation for Proteomics:*

Tissue specimens of approximately 15 mg were lysed using a Precellys 24 homogenizer (Bertin Technologies, Montigny-le-Bretonneux, France), ceramic beads, and 10 µl RIPA buffer per 1 mg tissue. After complete homogenization, samples were centrifuged at 14,000 g for 5 min, the supernatants were transferred to new tubes and stored at -80°C until further use.

Digestion was performed using single-pot, solid-phase enhanced sample preparation (SP3) (Hughes, C. S., S. Moggridge, T. Muller, P. H. Sorensen, G. B. Morin and J. Krijgsveld (2019). Single-pot, solid-phase-enhanced sample preparation for proteomics experiments. Nat Protoc 14(1): 68-85.) Briefly, the reduced (10 mM DTT for 1h at 56°C) and alkylated (55 mM IAA, 30 min at RT) proteins were bound to Sera-Mag beads (Merck, Darmstadt, Germany; 10:1 beads:protein ratio), washed with 80% ethanol and acetonitrile (ACN), and subjected to on-bead digestion with trypsin/LysC (Promega; 1:25 protease:protein ratio), overnight at 37°C in 50 mM ammonium bicarbonate, pH 8.5 (Sigma). Eluted peptides were desalted using Pierce Peptide Desalting Columns (Thermo Fisher Scientific, Waltham, MA, USA), dried in a vacuum concentrator and reconstituted in 100 mM TEAB, pH 8.5 (Fluka). Peptide concentration was determined using the Colorimetric Peptide Assay (Thermo Fisher) according to the protocol. Labeling with TMTpro reagents (Thermo Fisher) was performed according to the instructions provided by the manufacturer. In brief, TMTpro reagents were reconstituted with ACN and 75 µg of each sample were labeled with 250 µg TMTpro reagent. After incubation for 1 h at RT, the reaction was quenched by addition of 5% hydroxylamine (Sigma) in TEAB and incubation for 15 min at RT. Labeling efficiency was determined by LC-MS analysis of a pool of small aliquots of each sample using the Proteome Discoverer (v2.4.0.305, Thermo Fisher) software.

*Offline fractionation and LC-MS:*

Pooled samples were concentrated and desalted using Pierce Peptide Desalting Columns (Thermo Fisher). Eluates were dried in a vacuum concentrator and reconstituted in 20 mM ammonia formate buffer, pH 10 before fractionation at basic pH. Two-dimensional liquid chromatography was performed by reverse-phase chromatography at high and low pH. In the first dimension, peptides were separated on a Gemini-NX C18 (150 x 2mm, 3 µm, 110 A, Phenomenex, Torrance, USA) in 20 mM ammonia formate buffer, pH 10 and eluted over a 44 min gradient from 0% to 60% solvent B (0.1% formic acid in ACN) followed by 5 min at 100% solvent B at 50 µl/min using an Ultimate 3000 RSLC micro system (Thermo Fisher Scientific) equipped with a fraction collector. Thirty-six fractions were collected in a time-based manner (every 30s from min 11.5 to 57). Organic solvent was removed in a vacuum concentrator and samples were reconstituted in 0.1% trifluoroacetic acid.

Fractions were analyzed on an Ultimate 3000 RSLC nano coupled directly to an Orbitrap Exploris 480 with FAIMSpro (all Thermo Fisher). Samples were injected onto a reversed-phase C18 column (50 cm x 75 µm i.d., packed in-house) and eluted with a gradient of 4% to 38% solvent B over 94 min by applying a flow rate of 230 nl/min. MS scans were performed in the range from m/z 375–1650 at a resolution of 120,000 (at m/z = 200). MS/MS scans were performed choosing a resolution of 30,000 with the turboTMT mode for TMTpro reagents; normalized collision energy of 33%; isolation width of 0.7 m/z and dynamic exclusion of 90s. Two different FAIMS voltages were applied (-40V and -60V) with a cycle time of 1.5 s per voltage. FAIMS was operated in standard resolution mode with a static carrier gas flow of 4.6 L/min.

*Data analysis:*

The acquired raw MS data files were processed and analyzed using Proteome Discoverer (v2.4.0.305, Thermo Fisher). SequestHT was used as search engine and following parameters were chosen: database: Mus musculus (SwissProt, downloaded on 2024-01-26); enzyme: trypsin; max. missed cleavage sites: 2; static modifications: TMTpro (K and peptide N-terminus) and carbamidomethyl (C); dynamic modifications: oxidation (M), deamidation (N, Q), acetyl (protein N-terminus), Met-loss (M) and Met-loss+Acetyl (M); precursor mass tolerance: 10 ppm; fragment mass tolerance: 0.02 Da.

For reporter ion quantification the most intense m/z in a 20 ppm window around the theoretical m/z was used. Correction of isotopic impurities for reporter ion intensities was applied. Only unique peptides were used for quantification, which was based on S/N values with an average S/N threshold of 10. Normalization was based on total peptide amount and scaling mode on all average. Only peptides and proteins with FDR <0.01 are reported and single peptide IDs were excluded from the dataset.

Testing for differentially regulated proteins was performed using R (v4.2.0; http://www.r-project.org) and the R-package "limma", using a design without intercept (formula: 0 ~ treatment-group), and comparisons of interest were extracted using a contrast matrix. P-values were corrected for multiple hypothesis testing using the BH-method. Only proteins having 3 abundance values per treatment-group were used for analysis.

**Supplemental Table 1**

Raw quantitative data for proteins associated with wikipathways "Calcium regulation in cardiac cells (Rattus norvegicus)" (WP326). The Excel tab lists protein accession, gene, description, peptide count, and differential abundance metrics (log2 fold change, p-value, and FDR) for the indicated comparisons (DMD vs. WT, IVA vs. DMD, and IVA vs. WT).

**Supplemental Figure 1**

Pathway visualization of the "Calcium Regulation in Cardiac Cells (Rattus norvegicus)" pathway (Wikipathways ID: WP326). Panels (A) and (B) show the fold changes (FC) of gene expression mapped onto pathway nodes, with coloration reflecting the direction and magnitude of change (blue to red scale for log2FC values between -1 and 1). Panel (A) compares DMDmdx versus WT conditions, while Panel (B) compares IVA+DMDmdx versus DMDmdx. Nodes corresponding to significantly differentially expressed genes (FDR < 0.05) are highlighted with a thick border, enabling the identification of key molecular alterations in calcium regulation across the respective comparisons.

**Supplemental Figure 2**

Scaled protein abundance levels of key sarcoplasmic reticulum (SR) calcium-buffering proteins and Atp2c1. Individual bar plots show changes in protein abundance for (A) Sarcalumenin (Srl), (B) Calsequestrin-2 (Casq2), (C) Histidine-rich calcium-binding protein (Hrc), (D) Triadin (Trdn), (E) Aspartate-β-hydroxylase (Asph; peptide evidence (PE)=1, splice variant (SV)=2), (F) Aspartate-β-hydroxylase (Asph; PE=2, SV=1), and (G) Secretory Pathway Ca2+-ATPase (Atp2c1), included as requested by the reviewer. The y-axis represents scaled abundance. Error bars indicate the standard deviation of the mean. *FDR < 0.05, **FDR < 0.01.
